# Obol: Integrating Language and Meaning in Bio-Ontologies

**DOI:** 10.1002/cfg.435

**Published:** 2004

**Authors:** Christopher J. Mungall

**Affiliations:** HHMI Department of Molecular and Cellular Biology 142 Life Sciences Addition #3200 University of California Berkeley CA 94720-3200 USA

## Abstract

Ontologies are intended to capture and formalize a domain of knowledge. The ontologies comprising the Open Biological Ontologies (OBO) project, which includes the Gene Ontology (GO), are formalizations of various domains of biological knowledge. Ontologies within OBO typically lack computable definitions that serve to differentiate a term from other similar terms. The computer is unable to determine the meaning of a term, which presents problems for tools such as automated reasoners. Reasoners can be of enormous benefit in managing a complex ontology. OBO term names frequently implicitly encode the kind of definitions that can be used by computational tools, such as automated reasoners. The definitions encoded in the names are not easily amenable to computation, because the names are ostensibly natural language phrases designed for human users. These names are highly regular in their grammar, and can thus be treated as valid sentences in some formal or computable language.With a description of the rules underlying this formal language, term names can be parsed to derive computable definitions, which can then be reasoned over. This paper describes the effort to elucidate that language, called Obol, and the attempts to reason over the resulting definitions. The current implementation finds unique non-trivial definitions for around half of the terms in the GO, and has been used to find 223 missing relationships, which have since been added to the ontology. Obol has utility as an ontology maintenance tool, and as a means of generating computable definitions for a whole ontology.

The software is available under an open-source license from: http://www.fruitfly. org/~cjm/obol. Supplementary material for this article can be found at: http://www. interscience.wiley.com/jpages/1531-6912/suppmat.


**Comparative and Functional Genomics**
*Comp Funct Genom* 2004; **5**: 509–520.
Published online in Wiley InterScience (www.interscience.wiley.com). **DOI:** 10.1002/cfg.435

**Conference Paper**

### Obol: integrating language and meaning in bio-ontologies

Christopher J. Mungall*
*HHMI, Department of Molecular and Cellular Biology, Life Sciences Addition, University of California, Berkeley, CA 94729-3200, USA*

**Correspondence to: Christopher J. Mungall, University of California Berkeley, Dept. of Molecular and Cell Biology, 142 Life Sciences Addition #3200, Berkeley, CA 94720-3200, USA. E-mail: cjm@fruitfly.org*

Received: 1 November 2004
Revised: 2 November 2004
Accepted: 3 November 2004

### Abstract

**Ontologies are intended to capture and formalize a domain of knowledge. The ontologies comprising the Open Biological Ontologies (OBO) project, which includes the Gene Ontology (GO), are formalizations of various domains of biological knowledge. Ontologies within OBO typically lack computable definitions that serve to differentiate a term from other similar terms. The computer is unable to determine the meaning of a term, which presents problems for tools such as automated reasoners. Reasoners can be of enormous benefit in managing a complex ontology. OBO term names frequently implicitly encode the kind of definitions that can be used by computational tools, such as automated reasoners. The definitions encoded in the names are not easily amenable to computation, because the names are ostensibly natural language phrases designed for human users. These names are highly regular in their grammar, and can thus be treated as valid sentences in some formal or computable language. With a description of the rules underlying this formal language, term names can be parsed to derive computable definitions, which can then be reasoned over. This paper describes the effort to elucidate that language, called Obol, and the attempts to reason over the resulting definitions. The current implementation finds unique non-trivial definitions for around half of the terms in the GO, and has been used to find 223 missing relationships, which have since been added to the ontology. Obol has utility as an ontology maintenance tool, and as a means of generating computable definitions for a whole ontology.**

**The software is available under an open-source license from: http://www.fruitfly.org/~cjm/obol. Supplementary material for this article can be found at: http://www.interscience.wiley.com/jpages/1531-6912/suppmat. Copyright © 2004 John Wiley & Sons, Ltd.**

### Introduction

The Gene Ontology (GO) is a collection of three ontologies, partitioned into orthogonal domains: molecular function, biological process and cellular component [1,2]. GO is part of the Open Bio-Ontologies (OBO) project [3], which includes ontologies for other biologically relevant domains, such as anatomy, cell type [4], chemical compounds and phenotypic descriptors [5,6].

Ontologies in OBO consist of *terms*, which are used to describe biological data, such as gene products. Each term must have a name, which is a concise phrase capturing the meaning of the term; e.g. 'negative regulation of interleukin-2 biosynthesis', 'cardioblast differentiation' and 'small ribosomal subunit'. Terms may also have one or more *synonyms*.

Terms are interconnected via typed binary *relationships*, such as 'interleukin **is_a** cytokine' or 'small ribosomal subunit **part_of** mitochondrial ribosome'.

Text definitions precisely state the exact meaning of a term. Not all terms have text definitions. Text definitions are interpreted by users, not computers. An OBO term has little in the way of computable

Copyright © 2004 John Wiley & Sons, Ltd.

definitions, which makes it difficult to perform certain kinds of reasoning over the terms in the ontology [7,8].

It has been noted [7,9–11] that many term names are compositional, and indicate *implicit* relationships to terms, possibly in a different ontologies; e.g. 'cardioblast differentiation' in the biological process ontology has an implicit relationship to the term 'cardioblast' in the cell ontology, yet this relationship is absent from both ontologies; currently there are no explicit relationships between different ontologies in OBO. A computable definition of 'cardioblast differentiation' would by necessity reference 'cardioblast'.

The existence of composite terms leads to redundancy in both text definitions and relationships [12]. For example, the term 'cytokine' has redundant definitions embedded in the text definitions for 'cytokine metabolism' and 'cytokine biosynthesis'. The redundancy in relationships manifests itself as 'cytokine metabolism' and 'cytokine biosynthesis' being related via **is_a** to both 'protein metabolism' and 'protein biosynthesis'.

The compositional nature of many terms leads to an increase in the number of relationships and consequent increase in complexity of the ontology. This is particularly true of the GO; the term 'positive regulation of T-helper 2 cell differentiation' has 114 distinct paths through the relationships in the ontology to the root term. This complexity can have a negative impact on both users and curators, searching and maintaining the ontologies.

We could integrate OBO by providing computable definitions for all existing terms. This would bring benefits, such as the ability to reason over the ontology and automatically derive certain relationships. However, adding and maintaining these computable definitions is a significant undertaking, and it is unclear whether the benefits would outweigh the costs.

An alternative approach is to use implicit knowledge encoded in a term name to derive the intended meaning of the term. OBO term names exhibit a high degree of regularity in phrase structure, and in how that structure relates to meaning. In fact, OBO term names can be viewed as phrases written in a *formal language*, described using logical rules. The formal language of OBO term phrases is a subset of natural language, and it thus serves the dual purpose of specifying meaning for both human users and computers.

This paper describes the attempt:

- To elucidate the formal language underlying OBO, called *Obol*.
- To use this language to derive the meaning of existing OBO terms.
- To use these derived meanings to help manage the redundancy and complexity within OBO.

### Materials and methods

### Source ontologies

A subset of OBO was used for constructing and testing Obol. The system described here was constructed using the three GO ontologies, the biochemical ontology and the cell ontology [4]. These ontologies were downloaded on 24 July 2004. Currently there is neither a generic (non-species-specific) anatomy or protein family ontology in OBO.

### Tokenization

Each term in OBO has exactly one primary name, which is a string of characters containing a phrase or sequence of words, indicated here by W*. The first step in determining the implicit meaning of a term is *tokenizing* the term name string.

Tokenization breaks a term name phrase-string into an ordered sequence of word-strings. Each of these word-strings is treated as an atomic (non-decomposable) token. The phrase-string is split on white-space characters and non-alphanumeric characters. White-space characters are discarded, but non-alphanumeric characters (e.g. the hyphen character '-') are preserved and treated as special word tokens. For example:

$$W^*_{\mathrm{GO:0045085}} = (\text{negative, regulation, of, interleukin, `}-\text{', 2, biosynthesis})$$

$$W^*_{\mathrm{GO:0006412}} = (\text{protein, biosynthesis})$$

### Atomic vocabularies (AVs)

Each word token is matched against an *atomic* vocabulary (AV) of words. The AV contains words, not phrases. It thus overlaps and is distinct from the

OBO-controlled vocabulary of phrases, where each phrase consists of one or more words.

The atomic vocabulary assigns each word a *domain*, and a *lexical category* (also known as a *part of speech*). The *domains* typically correspond to the domains of existing ontologies within OBO (e.g. molecular_function, cellular_component, cell, biochemical) and also to domains that are not presently covered by OBO (environment, protein, generic anatomy). Table 1 shows a list of domains defined by Obol.

The *lexical category* of a word defines the role that word plays in a phrase. For example, in the phrase 'negative regulation', the word 'negative' plays the role of adjective. For simplicity we assume that each word in the AV has exactly one lexical category. The lexical categories used include typical linguistic categories, such as nouns, adjectives, prepositions and relational adjectives. Relational adjectives are useful for linking adjectives to the noun form of that adjective. They are treated as binary relations between words; e.g. **relational_adj(*epidermal, epidermis*)**. There is also an additional category for numbers, roman numerals, words designating Greek symbols and alphanumeric characters used as 'type designators'; e.g. 'myosin II', 'interferon-alpha' and 'interleukin-2'.

**Table 1.** The 13 domains used to partition the Atomic Vocabularies. Some ontologies have been split into more than one domain; this was necessary if the ontology did not contain complete **is_a** parentage to a suitable upper-level term

| Domain | Corresponding OBO ontology | Notes |
|---|---|---|
| General | — | Mostly prepositions |
| Anatomy | Various species-specific | Generic anatomical structures |
| Function | GO molecular_function | |
| Process | GO biological_process | |
| Component | GO cellular_component | Cell parts |
| Cell | Cell | Cell types |
| Biochemical | Biochemical | Chemical compounds |
| Protein | *In progress* | e.g. Keratin, actin, interleukin-2 |
| Environment | — | e.g. Taste, touch, light |
| Behaviour | GO biological_process | Behaviour-specific processes |
| Enzyme | GO molecular_function | e.g. Amylase, deaminase |
| Organism | — | e.g. Viral/virus, bacteria, parasite |
| Sequence | Sequence | e.g. Transcript, five, prime |

OBO terms typically do not contain verbs, so there is no need for such a category. Inflected verbs (e.g. 'regulation', the inflected form of 'regulate') are treated no differently from other nouns. It is rare for OBO term names to include the definite or indefinite article ('the' or 'a'), and other lexical categories commonly used in natural language ('it', 'they', 'he', 'she'). It is also rare for there to be different variants of the same word, so there is no requirement for reducing words to stem forms.

The phrase 'negative regulation of interleukin-2 biosynthesis' can be tokenized into words that are categorized into the following lexical-categories and domains:

- **negative** (adjective, general).
- **regulation** (noun, biological_process).
- **of** (preposition, general).
- **interleukin** (noun, protein_family).
- - *(hyphen)* (special token).
- **2** (type designator, general).
- **biosynthesis** (noun, biological_process).

### Constructing the atomic vocabulary

All term names in the source ontology were tokenized; this resulted in a set of word tokens, which comprised the initial AV. Domains and lexical categories were assigned both manually and semi-automatically, using the OBO *grammar* (see next section).

As OBO changes, the AVs must also change. However, the atomic vocabularies need not be completely up-to-date, because the system exhibits graceful degradation with incomplete or incorrectly categorized AVs; performance is impacted, but not severely.

### OBO term grammar

A computational (or formal) grammar is a way to describe a formal language, analogous to the concept of grammars for natural languages [13,14]. A formal language is a set of sequences (e.g. sentences) over a finite alphabet (e.g. words).

Copyright © 2004 John Wiley & Sons, Ltd. 

A computational grammar $G$ consists of:

- A finite set $\Sigma$ of *terminal* symbols.
- A finite set $N$ of *non-terminal* symbols, disjoint from $\Sigma$.
- A finite set $P$ of production rules, where a rule is of the form:
  — Some string in $(\Sigma \cup N)^* \rightarrow$ some string in $(\Sigma \cup N)^*$.
  — [where $(\Sigma \cup N)^*$ indicates zero or more occurrences of terminal and non-terminal symbols].
- A symbol $S$ in $N$ that is indicated as the start symbol.

The language of a formal grammar $G = (N, \Sigma, P, S)$, denoted as $\mathbf{L}(G)$, is defined as all those strings over $\Sigma$ that can be generated by starting with the start symbol $S$ and then applying the production rules $P$ until no more non-terminal symbols are present.

A grammar can be used for either generating or parsing sequences of tokens. Parsing a sequence of tokens with a grammar will produce a parse tree, which can be used to elucidate the structure of the sequence. A sequence of tokens may have zero or more parse trees.

OBO term names are both natural language phrases and well-formed phrases conforming to a formal grammar.

The formal grammar $G_{OBO}$ contains terminal symbols $\Sigma_{OBO}$ equivalent to the word tokens from the AVs. The set of non-terminal symbols $N_{OBO}$ is the union of the set of lexical categories (nouns, adjectives, etc.) and the set of *phrase types* used to construct a term. Examples of phrase types are *noun phrases* and *prepositional phrases*.

The start symbol $S_{OBO}$ refers to a complete OBO term name.

The production rules $P_{OBO}$ specify how larger phrases are recursively constructed from smaller phrases and from words. A simplified subset of the production rules is included below; the entire grammar can be viewed by downloading the system. Non-terminal symbols are indicated with a leading upper-case character, terminal symbols in lower-case:

- **$S_{OBO}$ → NounPhrase**
  (an OBO term name is a noun phrase; e.g. the noun phrase 'negative regulation of protein biosynthesis')
- **NounPhrase → Noun**
  (a noun phrase can be a single noun; e.g. 'regulation' or 'biosynthesis')
- **NounPhrase → *Adjective*NounPhrase**
  (a noun phrase can be an adjective immediately followed by a noun phrase; e.g. '*negative* regulation' or '*smooth* muscle contraction')
- **NounPhrase → NounPhrase' – ' *Token***
  (a noun phrase can be a noun phrase immediately followed by a type designator; e.g. 'myosin *II*' or 'interleukin-*2*' or 'interferon-*alpha*')
- **NounPhrase → *NounPhrase* NounPhrase**
  (a noun phrase can be a stem noun phrase immediately preceded by a modifier noun phrase; e.g. '*interleukin-2* biosynthesis' or '*muscle* contraction'). Note that this rule is *left-recursive*, which can cause problems with some computational systems.
- **PrepPhrase → *Prep* NounPhrase**
  (a prepositional phrase can be a noun phrase immediately preceded by a preposition; e.g. '*of* interleukin-2 biosynthesis' or '*by* pheromones')
- **NounPhrase → NounPhrase *PrepPhrase***
  (a noun phrase can be a noun phrase immediately followed by a prepositional phrase; e.g. 'negative regulation *of interleukin-2 biosynthesis*')
- **Noun → abscission|absorption|accumulation|acetylation**|...
  **Adjective → apical|basal|early|endocytic**|...
  **Prep → by|of|in|as|during|via|with|using**|...
  **Token → 1|2**|...|**alpha|beta**|...|**A|B**|...|**I|II|III|IV**|...
  (lexical categories map to words in the AVs. Note the use of the pipe symbol '|' to indicate 'or')

Not shown here are rules for dealing with relational adjectives (e.g. 'cytosolic ribosome') and for Boolean connectors (e.g. 'recognition *and* cleavage').

$G_{OBO}$ production rules typically have one symbol on the left-hand side and one or two symbols on the right-hand side. When a rule has two symbols on the right-hand side, one symbol acts as the *stem* phrase, the other as the *supplementary* phrase. In the example rules given above, supplementary phrases are indicated with italics.

An example syntax parse of the GO term with name 'negative regulation of interleukin-2 biosynthesis' is shown in Figure 1. Stem phrases are indicated with the bold lines.

Copyright © 2004 John Wiley & Sons, Ltd.

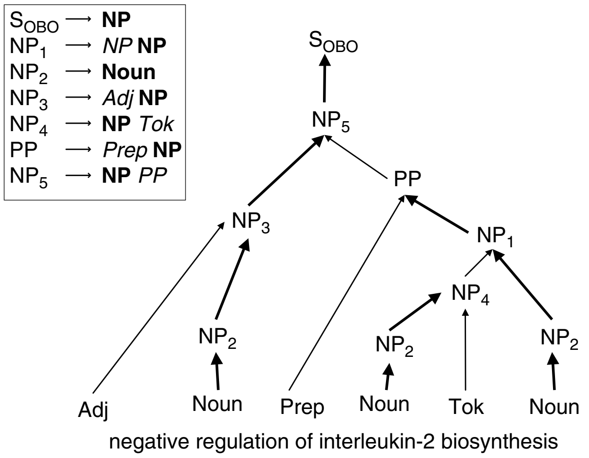

**Figure 1.** An example parse of a GO term. Stem phrases are indicated by bold text

The syntax parse can also be shown as a bracketed expression. There are actually two possible parses under $G_{OBO}$:

1. (NP(NP negative$_{adj}$ regulation$_{noun}$)
   (PP of$_{prep}$(NP(NP interleukin$_{noun}$ — 2$_{token}$)
   biosynthesis$_{noun}$)))
2. (NP negative$_{adj}$
   (NP regulation$_{noun}$
   (PP of$_{prep}$(NP(NP interleukin$_{noun}$ — 2$_{token}$)
   biosynthesis$_{noun}$))))

$P_{OBO}$ is augmented with *precedence rules* (not shown) to favour the first parse.

$P_{OBO}$ was constructed manually. Not all GO term names are in $\mathbf{L}(G_{OBO})$, which is to say that not all term names can be parsed. Some term names have more that one possible parse tree, indicating possible ambiguity in the syntactic structure (and thus in the interpreted meaning of the term).

$\mathbf{L}(G_{OBO})$ contains an infinite amount of potential term names, a finite subset of which correspond to meaningful biological phrases. The next step is to derive these meaningful phrases.

### Deriving class definitions

The grammar $G_{OBO}$ specifies a *syntactic* parse of OBO terms; this elucidates the compositional structure of term names. The next step is to derive a *semantic* parse; in other words, to derive the meaning encoded in a term name. The meaning of a term can be formally specified as an *Obol definition*, modelled after Aristotelian definitions [15].

An Obol definition consists of a *genus* and *differentiae*. The genus is a broad category (as distinct from genus, *sensu* phylogeny). Obol considers each word in the AV to be a distinct genus. The differentiae are a set of necessary and sufficient conditions that distinguish a term from other terms of the same genus. The differentiae are similar to standard relationships between OBO terms. However, they are different in that these standard relationships are not always sufficient to distinguish one term from other terms of the same genus. For example, the term 'interleukin-2 biosynthesis' is defined as having genus 'biosynthesis' and the differentia '**forms** interleukin-2'. The property of creating interleukin-2 proteins is sufficient to discriminate this term from all other kinds of biosynthesis.

Definitions can be nested. The definition for 'interleukin-2' would have genus 'interleukin' and differentia '**type_token** 2'. Using the notation <**genus**><***rel − type***> = (<**definition**>) to specify a definition, and simply <**genus**> for a primitive definition. Figure 2 shows the definitional structure of the GO term 'negative regulation of interleukin-2 biosynthesis'.

Genus–differentiae definitions are useful for automated reasoning. For example, we can prove that 'interleukin-2 biosynthesis' **is_a** 'interleukin biosynthesis' **is_a** 'cytokine biosynthesis' from a single relationship 'interleukin' **is_a** 'cytokine', given some ontology of protein families.

It is possible to derive candidate definitions for phrases using a grammar, $G$, a set of **is_a** relationships, $R$, ranging over the genus categories in an AV, and set of biological relationship types, $Q$. OBO has minimal set of logical relations defined in the relations ontology [16]. This was manually

**(regulation *qualifier* = (negative)**
***affects* = (biosynthesis *forms* = (interleukin *type_ token* = (2))))**

**Figure 2.** Class definition for the term 'negative regulation of interleukin-2 biosynthesis'

Copyright © 2004 John Wiley & Sons, Ltd.

**Table 2.** Examples of how grammatical contexts can be used to derive which relationship forms the differentia in a definition. Example terms are provided, with the genus shown in bold and the differentia term shown in italics. Note that this is just a subset of the contexts — the relationship 'part_of' can be part of a set of differentiae in a variety of contexts. Currently the only relationship type above defined by GO is 'part_of', Obol has to extend this basic set in order to derive definitions

| Relationship type | Domain | Range | Grammatic context | Example (genus *differentia*) |
|---|---|---|---|---|
| Qualifier | Regulation | −ve OR + ve | Adjective prefix | *Negative* **regulation** |
| Affects | Process | Process | Prep phrase with 'of' | **Regulation** of *xxx biosynthesis* |
| Forms | Biosynthesis | Substance | Noun phrase prefix | *Interleukin-2* **biosynthesis** |
| Type_token | Any | Token | Type token | **Interleukin**-*2* |
| Part_of | Component | Component | Relational adjective | *Cytoplasmic* **chromosome** |

**Table 3.** Some results from applying semantic parses on typical OBO terms. Recursive definitions are shown as successive parses of each differentia term. Note that there is no protein ontology in OBO at present, so Obol creates a temporary ID for terms such as 'interleukin-2'. Note also that there is no GO term for the genus 'regulation'

| OBO ID | Term name | Genus | Differentiae |
|---|---|---|---|
| GO:0045085 | Negative regulation of interleukin-2 biosynthesis | Regulation | Qualifier = negative affects = GO:0042094 |
| GO:0042094 | Interleukin-2 biosynthesis | Biosynthesis (GO:0009058) | Forms = <tempID:1> |
| <tempID:1> | Interleukin-2 | Interleukin | Type_token = 2 |
| GO:0000229 | Cytoplasmic chromosome | Chromosome (GO:0005694) | Part_of = GO:0005737 (cytoplasm) |

augmented to get $Q_{OBO}$. Here, a relationship type belonging to $Q$ is defined by a set of *domain, range* and *grammatical context* triples. The domain and range specify to which genus or genera a relationship type pertains. The grammatical context specifies how a relationship is linguistically manifested in a phrase. For example, consider the relationship type **affects** in $Q_{OBO}$. This type can have the domain 'regulation' and the range 'process' occurring with the preposition 'of' in some phrase. Because 'biosynthesis' **is_a** 'process', the phrase 'regulation of biosynthesis' is equivalent to the definition **regulation *affects* = (biosynthesis)**. Table 2 shows some example relationships and the contexts in which they are used.

OBO term names are remarkably consistent in mapping grammatical structure to meaning. A term with name as 'regulation of transcription' would never use a different grammatical context (except as a synonym). An example of a different grammatical context is a relational adjective modifier, as in 'transcriptional regulation'.

### Class definition derivation method

Given a set of relationship types $Q$, as defined above, we can attempt to infer a class definition for any syntax parse tree.

Starting at the root of the parse tree, S, we recursively build the class definition. The production rules, $P$, are constructed such that each node in the parse tree should have one or two children. If it has one child, then the class definition at the parent node is equal to the class definition at the child node. If the node has two children — one stem node $n_1$ and another supplementary node $n_2$ — then the class definition will be the combination of the class definition $D_1$ at $n_1$ with a relationship, $r$, to the class definition $D_2$ at $n_2$, where the possible relationship types are constrained by the grammatical type of the current node, $n$, together with the domain and range types matched to $D_1$ and $D_2$, according to $Q$.

When we reach the leaf nodes of the parse tree, we assign a *primitive definition*, which is simply a genus lacking differentiae.

Copyright © 2004 John Wiley & Sons, Ltd.

Deriving a class definition is also known as *semantic parsing*. This extension to a standard grammar is called a *semantic grammar*. A semantic grammar is reversible, allowing for the generation of phrases from definitions.

Table 3 gives an example of the semantic parse of the term name 'negative regulation of interleukin-2 biosynthesis'.

If we augment our definition of a grammar $G$ with relationship types and a set of **is_a** relationships over genera, giving a semantic grammar $\Gamma = (N, \Sigma, P, S, Q, R_{IS_A})$, then the language of $\Gamma$, denoted as $\mathbf{L}(\Gamma)$, is defined as all those *meaningful* strings over $\Sigma$ belonging to $\mathbf{L}(G)$. If $\Gamma_{\mathrm{OBO}}$ is defined perfectly, then $\mathbf{L}(\Gamma_{\mathrm{OBO}})$ should correspond to the universe of meaningful biological terms. We refer colloquially to $\mathbf{L}(\Gamma_{\mathrm{OBO}})$ as *Obol*.

Definitions can be exported using either the obo flat-file format (**http://www.geneontology.org/GO.format.html#oboflat**) or using a description logic (DL) format, such as Ontology Web Language (OWL) [17].

The candidate class definitions can be considered an end in and of themselves, or they can be used for reasoning.

### Reasoning

Class definitions can be used for reasoning over the ontologies. This can be done using an external third-party reasoner, such as FaCT [18] or RACER [19]. There is also an advantage to integrating some simple reasoning facility into the grammar system.

Some of the things we may wish to do with a reasoner and some class definitions include:

- Check for inconsistent or missing relationships, or missing terms.
- Automatically assign relationships for new terms.
- Extract implicit ontologies from GO.

The reasoner can also be used to feed back information to the class builder, to help filter ambiguous parses.

The Obol reasoner uses a rule-based system for performing these tasks. An example of a simple Obol rule is one involving subsumption (subtyping). Here the notation **is_a**$^+$ means transitive **is_a**, and T **is_a**$^+$ T$'$ means that T$'$ can be reached from T by following a chain of one or more **is_a** relationships.

T **is_a**$^+$T$'$ *if* (T **is_a** X and X **is_a**$^+$T$'$) *or* (T **is_a** T$'$)

There are rules for inferring contingent inclusion **is_a** relationships [16] that are based on natural **is_a** relationships using genus–differentiae definitions (where G = genus, and the differentia is Q = (D), and D is itself a definition). Here is a subset of the Obol rule-set:

1: G,Q = (D) **is_a** G,Q = (D$'$) *if* D **is_a** D$'$
2: G,Q = (D) **is_a** G$'$, Q = (D) *if* G **is_a** G$'$
3: G,Q = (D) **is_a** G *if* $\neg(\exists$D$'$ *such that* D **is_a** D$')$

Using rule 1, and the semantic grammar, the Obol system infers an **is_a** relationship between 'chromoplast membrane' (**membrane *surrounds* = chromoplast**) and 'plastid membrane' (**membrane *surrounds* = plastid**) based on the **is_a** relationship between 'chromoplast' and 'plastid'.

An example of applying rule 2 is to discover that 'vitamin E biosynthesis' (**biosynthesis *forms* = vitaminE**) **is_a** 'vitamin E metabolism' (**metabolism *forms* = vitaminE**), based on the **is_a** relationship between 'biosynthesis' and 'metabolism'.

An example of applying rule 3 is to discover that 'primary septum' (**septum *type* = primary**) **is_a** 'septum', based on the fact that there is no OBO term 'primary' and thus no parent for that term.

Rules such as these can be used to find errors of omission in an ontology, to automatically add relationships for newly created terms, to suggest intermediate terms, and to determine which relationships in the ontology are true by contingency.

Table 4 has the complete set of all relationships that can be derived for the term named 'negative regulation of interleukin-2 biosynthesis'. Note that all these derived relationships are currently present in GO due to curator diligence.

### Implementation

The grammar, class builder and reasoner, described above, are all specified and implemented in Prolog. The grammar is implemented as a Definite Clause Grammar (DCG) [20]. Prolog allows for direct specification of DCGs as part of the language. Prolog is a high-level declarative logic programming language, and is considerably different from

Copyright © 2004 John Wiley & Sons, Ltd. 

**Table 4.** Examples of relationships that can be automatically derived by semantically parsing the term 'negative regulation of interleukin-2 biosynthesis'. Note that all 16 of these relationships are currently present in GO, so Obol tells us nothing new here. However, manual curation of these derivable relationships is an onerous and error-prone task. Obol can help maintain these. Note also that in order to make all of these derivations Obol requires an ontology of proteins, such as interleukins. No such ontology currently exists within OBO — an example of one was generated for this particular test. This highlights the importance of OBO for the GO project

| Child term | Relationship type | Parent term | Notes |
|---|---|---|---|
| Negative **regulation** of interleukin-2 biosynthesis | Is_a | **Regulation** of interleukin-2 biosynthesis | 'Negative' has no parents |
| | Is_a | Negative **regulation** of cytokine biosynthesis | From: interleukin-2 is_a cytokine |
| **Regulation** of interleukin-2 biosynthesis | Is_a | **Regulation** of cytokine biosynthesis | From: interleukin-2 is_a cytokine |
| | Regulates | Interleukin-2 **biosynthesis** | Via inferred definition |
| Negative **regulation** of cytokine biosynthesis | Is_a | **Regulation** of cytokine biosynthesis | 'Negative' has no parents |
| | is_a | Negative **regulation** of protein biosynthesis | From: cytokine is_a protein |
| **Regulation** of cytokine biosynthesis | Is_a | **Regulation** of protein biosynthesis | From: cytokine is_a protein |
| | Regulates | Cytokine **biosynthesis** | Via inferred definition |
| Negative **regulation** of protein biosynthesis | Is_a | Negative **regulation** of biosynthesis | 'Protein' is root term in protein ontology |
| | Is_a | **Regulation** of protein biosynthesis | 'Negative' has no parents |
| **Regulation** of protein biosynthesis | Is_a | **Regulation** of biosynthesis | 'Protein' is root term in protein ontology |
| | Regulates | Protein**biosynthesis** | Via inferred definition |
| **Regulation** of biosynthesis | Regulates | **Biosynthesis** | Via inferred definition |
| Interleukin-2 **biosynthesis** | Is_a | Cytokine **biosynthesis** | From: interleukin-2 is_a cytokine |
| Cytokine **biosynthesis** | Is_a | Protein **biosynthesis** | From: cytokine is_a protein |
| Protein **biosynthesis** | Is_a | **Biosynthesis** | 'Protein' is root term |

**Table 5.** An illustrative subset of the 400 purported missing relationships derived by the system. Some of these derived missing relationships were incorrect, based on erroneous definition parses. The final example illustrates a case where the formal definition of a term cannot be derived from the term phrase alone

| Subject | Relationship type | Object | Notes |
|---|---|---|---|
| Nucleolar chromatin | Part_of | Nucleolus | Derived directly from inferred definition |
| Clathrin-coated vesicle | Has_part | Clathrin coat | Correct, but GO uses **part_of** |
| Chromoplast membrane | Is_a | Plastid membrane | Derived via 'chromoplast is_a plastid' |
| Nuclear microtubule | Part_of | Nucleus | Derived directly from inferred definition |
| Vitamin E biosynthesis | Is_a | Vitamin E metabolism | From: biosynthesis is_a metabolism |
| Negative regulation of lipid biosynthesis | Is_a | Negative regulation of lipid metabolism | From: biosynthesis is_a metabolism |
| Dense nuclear body | Is_a | Nuclear body | INCORRECT |

Copyright © 2004 John Wiley & Sons, Ltd. 

languages typically used in bioinformatics, such as perl, C and java. It is particularly well suited to implementing logical rules, the foundations of the Obol system. We use XSB Prolog [21], which provides tabling in addition to a standard prolog system. This allows grammar production rules to be left-recursive, as is the case for the $P_{OBO}$. The go-perl library [22] is used to convert OBO files to prolog fact files. Interaction with the system is either via prolog interpreter or via a UNIX makefile. The system has been tested on both Linux and Mac OS X, and is available under an open source licence from **http://www.fruitfly.org/~cjm/obol**.

### Results and discussion

### Parsing

Any OBO term can have zero, one or multiple ambiguous syntactic or semantic parses. We tested the system on all terms in GO (but not the rest of OBO). Ideally, one semantic parse (class definition) would be generated for each GO term, but in practice this is currently only achieved for about half of all terms. Ideally, the semantic parse would reflect the actual meaning of the term, but in some cases inaccurate meanings are derived.

Typically, term names with lots of words generate multiple ambiguous parses. In particular, term names that contain long sequences of nouns result in an exponential increase in syntactic parses; this is due to the rule **NounPhrase → NounPhrase NounPhrase**. Note that it is still possible to do reasoning with ambiguous parses.

The histograms in Figures 3, 4 and 5 show the distribution of the number of parses, both syntactic and semantic, for the three GO ontologies. Single-word terms have been excluded, since these are trivially parsed to the corresponding genus. The histograms have been truncated at five parses; one particular molecular function term ('receptor signaling protein tyrosine kinase signaling protein activity') has 132 syntactic parses (of which none derive a definition).

Note that the molecular function ontology has the highest number of terms that cannot be parsed. One reason is that the tokenizer and grammar originally did not deal adequately with term names containing chemical notation, such as '5($S$)-hydroxyperoxy−6$E$, 8$Z$, 11$Z$, 14$Z$-icosatetraenoic acid binding'. This has recently been fixed by adding tokenizer rules, such that compound identifiers are treated as single tokens.

Improving the AVs will reduce the number of incorrect syntax parses (and thus reduce the incorrect semantic parses). Many words are incorrectly categorized or are not yet present in the AVs. This can be improved by manual and semi-automatic AV curation.

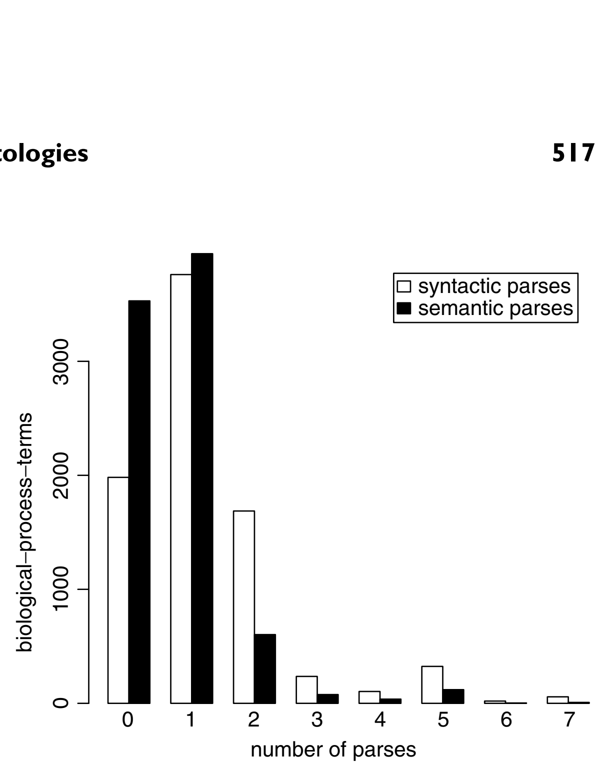

**Figure 3.** The distribution of the number of parses, both syntactic and semantic, for the biological process GO ontology

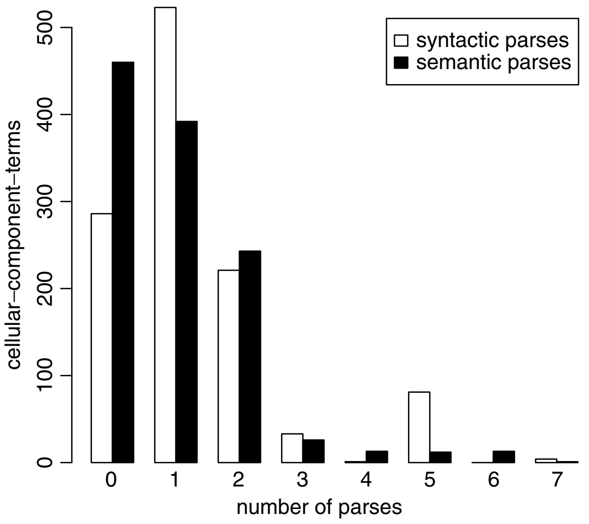

**Figure 4.** The distribution of the number of parses, both syntactic and semantic, for the cellular component GO ontology

Copyright © 2004 John Wiley & Sons, Ltd.

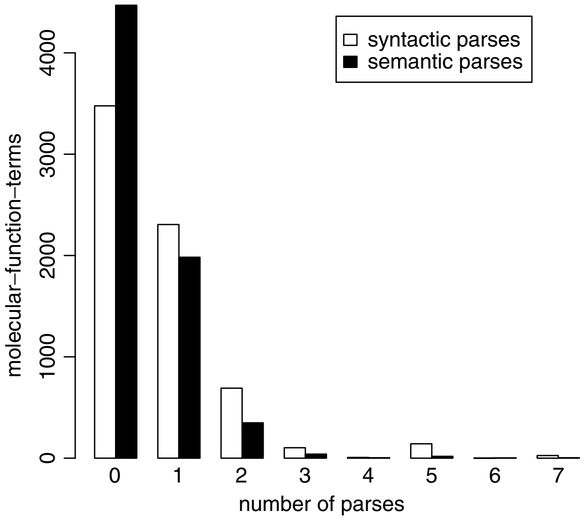

**Figure 5.** The distribution of the number of parses, both syntactic and semantic, for the molecular function GO ontology

Refining the set of relationship types will improve semantic parsing. Currently only very general relationship types are used. More specific relationship types could help filter out ambiguous parses, and provide more specific meanings.

### Exporting definitions

The derived definitions can be represented as an OWL file. An export of all of the source ontologies is available as Online Supplementary Material at: **http://www.interscience.wiley.com/jpages/1531-6912/suppmat**. This file was generated automatically. An OWL definition is provided only if there is exactly one semantic parse for a term.

### Missing relationships

We found 400 candidate missing relationships over two iterations. The first run was performed on the March 2004 version of GO, with an earlier version of the code; the second run was performed on the July 2004 version of GO. Of these 400, at least 223 have since been added to GO. Each term was parsed to derive a class definition. Reasoning rules were applied over the class definitions to infer missing relationships. Table 5 shows some examples of the missing relationships detected.

### Deriving existing relationships

We examined all 23 306 relationships in GO to determine which could be automatically derived. We compared two methods. The first method uses the semantic grammar $\Gamma_{OBO}$ and relationship derivation rules, as above. The second method is similar to that described in [10], in which a relationship is derivable if all the words in the parent term are an ordered subset of words in the child term. Table 6 shows the numbers of derivable relationships in GO. This gives a rough indication of what proportion of GO relationships reflect biological knowledge, and what proportion are true by their logical definition.

Use of a semantic grammar has lower sensitivity than a subset- or substring-based approach. This is because many terms cannot be parsed by $\Gamma_{OBO}$, The sensitivity will improve as $\Gamma_{OBO}$ improves. It is difficult to measure specificity, because GO relationships may be incomplete.

### Generating implicit ontologies

The GO biological process ontology contains an implicit generic anatomy ontology, encoded in term names such as 'haltere disc metamorphosis' and corresponding relationships. This implicit anatomical ontology has been extracted, and could be used as the basis for a cross-species generic anatomy.

### Limitations

Not all composite term names reflect the exact meaning of the term. For example, 'dense nuclear body' refers to something more specific than what is suggested by the individual words. This can lead to errors in reasoning, such as 'dense nuclear body' **is_a** 'nuclear body'. This can be overcome by manually creating formal definitions that override

**Table 6.** Derivable relationships in GO. Two methods are compared, one using Obol, and the other using a subset relation between words in the two term phrases

| | Function | Process | Component |
|---|---|---|---|
| Total relationships | 8002 | 13 613 | 1691 |
| Obol-D | 479 (6%) | 3055 (22%) | 346 (20%) |
| Subset-D | 2135 (27%) | 4299 (32%) | 534 (32%) |
| Obol-D, Subset-ND | 0 | 1089 (8%) | 63 (3%) |
| Obol-ND, Subset-D | 1656 (21%) | 2333 (17%) | 251 (15%) |

D, derivable; ND, non-derivable.

Copyright © 2004 John Wiley & Sons, Ltd.

the derived definition. Similarly, there are many words that Obol incorrectly treats as primitive definitions lacking differentiae. Examples include ‘chemotaxis’ and ‘neurogenesis’.

### Future directions

OBO terms occasionally use concise colloquial term names rather than term names that explicitly indicate the meaning according to $\Gamma_{OBO}$. These terms typically have an exact synonym. Future implementations will also parse exact synonyms. Another simple extension would allow for automatic generation of synonyms for terms.

Text definitions also conform to a regular structure, and so it should be possible to decompose and generate these. However, these are less concise than term names, and have a more complex grammar. A different approach is required for parsing text definitions.

The main benefit of using Obol is as an aid to the curation process, in that they assist with deriving relationships. In this mode, the end-user of the ontology would not use the grammar in any way. However, it is in theory possible to use Obol to enhance searching and navigation. For example, if a user searches for genes involved in ‘transcriptional regulation’, Obol could map this onto the correct term name ‘regulation of transcription’. Other end-users who may benefit from the richer definitions that come from parsing include bioinformatics users who use measures of semantic similarity [23].

While it is important to stress that parsing a highly restrictive language (such as the language of OBO terms) is much simpler than parsing natural language, it may be possible to use parts of $\Gamma_{OBO}$ as an aid to natural language processing. An example application is deriving the meaning of sentences in Medline abstracts. Another possibility is alternative grammars and lexical mappings to aid automatic translation of OBO term names to languages other than English.

The software described here was designed with the needs of the OBO community in mind. However, much of it could be applied to ontologies that are not part of OBO, biological or otherwise. This would only be worthwhile if the target ontology contained a large number of compound terms, with the implicit knowledge encoded in highly regular term names not reflected in the term definitions. Different domains may require alterations to the grammar production rules, as well as their own AVs and relationship types; however, the core parts of the Obol software could be used unaltered.

One of the most intriguing possibilities offered by Obol is to assist in the permanent manual transition of OBO from the existing representation to a more formal representation, such as a genus–differentiae paradigm as outlined here, or a description logic [7] paradigm. Such a transition would be extremely daunting without computational assistance. This would mark a significant change in OBO curation, but could have significant benefits for curators in terms of time spent creating and maintaining compound terms.

### Acknowledgements

This work was supported by the Howard Hughes Medical Institute, and the Gene Ontology Consortium P41 Grant from the National Human Genome Research Institute (NHGRI), Grant No. HG002273. I am grateful to many colleagues for their help with this research, in particular to Midori Harris, Jennifer Clark, Amelia Ireland and Jane Lomax for evaluating the initial results, and to Suzanna Lewis, John Day-Richter, David Hill and Michael Ashburner for their insightful comments, ideas, inspiration and encouragement.

### References

1. Harris MA, *et al.* 2004. The Gene Ontology (GO) database and informatics resource. *Nucleic Acids Res* **32**: (database issue): D258–261.
2. Ashburner M, *et al.* 2000. Gene ontology: tool for the unification of biology. The Gene Ontology Consortium. *Nature Genet* **25**(1): 25–29.
3. Open Biological Ontologies: **http://obo.sourceforge.net/**
4. Bard J, Reed SY, Ashburner M. 2004. An ontology for cell types. *Genome Biology* (manuscript submitted).
5. Drysdale R. 2001. Phenotypic data in FlyBase. *Brief Bioinform* **2**(1): 68–80.
6. Gkoutos GV, *et al.* 2004. Building mouse phenotype ontologies. *Pac SympBiocomput* 178–189.
7. Wroe CJ, *et al.* 2003. A methodology to migrate the gene ontology to a description logic environment using DAML + OIL. *Pac Symp Biocomput* 624–635.
8. Smith B, Williams J, Schulze-Kremer S. 2003. The ontology of the gene ontology. *AMIA Annu Symp Proc* 609–613.

Copyright © 2004 John Wiley & Sons, Ltd.

9. Hill DP, *et al*. 2002. Extension and integration of the gene ontology (GO): combining GO vocabularies with external vocabularies. *Genome Res* **12**(12): 1982–1991.
10. Ogren PV, *et al*. 2004. The compositional structure of Gene Ontology terms. *Pac Symp Biocomput* 214–225.
11. Yeh I, *et al*. 2003. Knowledge acquisition, consistency checking and concurrency control for Gene Ontology (GO). *Bioinformatics* **19**(2): 241–248.
12. Smith B, Köhler J, Kumar A. 2004. On the application of formal principles to life science data: a case study in the Gene Ontology. In *Data Integration in the Life Sciences (DILS)*: 79–94.
13. Chomsky N. 1959. On certain formal properties of grammars. *Inform Control* **2**: 137–167.
14. Formal grammar. In *Wikipedia: The Free Encyclopedia*. Vol. 8, Sept. 2004.
15. Cohen S.M. 2003. Artistotle's metaphysics. In *The Stanford Encyclopedia of Philosophy*, Zalta EN (ed.).
16. Smith B, WC, Köhler J, Kumar A, *et al*. 2004. Relations in biological ontologies. *Genome Biology*, submitted.
17. OWL Web Ontology Language: **http://www.w3.org/TR/2004/REC-owl-ref-20 040 210/**
18. Horrocks I. 1998. The FaCT system. In *Automated Reasoning with Analytic Tableaux and Related Methods: International Conference Tableaux '98* 307–312.
19. Volker Haarslev RM. 2003. Racer: a core inference engine for the semantic web. In *Proceedings of the 2nd International Workshop on Evaluation of Ontology-based Tools (EON2003)*. Sanibel Island, FL, USA.
20. Clocksin WF, Mellish CS. 1981. *Programming in Prolog*. Springer-Verlag: New York.
21. XSB Prolog: **http://xsb.sourceforge.net**
22. go-perl library: **http://www.godatabase.org/dev/go-perl/doc/go-perl-doc.html**
23. Lord PW, *et al*. 2003. Investigating semantic similarity measures across the Gene Ontology: the relationship between sequence and annotation. *Bioinformatics* **19**(10): 1275–1283.

Copyright © 2004 John Wiley & Sons, Ltd. 
